# The Lazy Visual Word Form Area: Computational Insights into Location-Sensitivity

**DOI:** 10.1371/journal.pcbi.1003250

**Published:** 2013-10-03

**Authors:** Thomas Hannagan, Jonathan Grainger

**Affiliations:** Laboratoire de Psychologie Cognitive, CNRS & Aix-Marseille University, Marseille, France; Brain and Spine Institute (ICM), France

## Abstract

In a recent study, Rauschecker et al. convincingly demonstrate that visual words evoke neural activation signals in the Visual Word Form Area that can be classified based on where they were presented in the visual fields. This result goes against the prevailing consensus, and begs an explanation. We show that one of the simplest possible models for word recognition, a multilayer feedforward network, will exhibit precisely the same behavior when trained to recognize words at different locations. The model suggests that the VWFA initially starts with information about location, which is not being suppressed during reading acquisition more than is needed to meet the requirements of location-invariant word recognition. Some new interpretations of Rauschecker et al.'s results are proposed, and three specific predictions are derived to be tested in further studies.

## Author Summary

There is a mild form of modern “mind-reading” that involves, with heavy fMRI apparatus and software assistance, to guess from brain signals alone the locations of words that have been seen by a (consenting) subject. The recent surprise brought to us by Rauschecker et al. is not that we can currently do that, but that we can do it in a brain region that had until now been largely taken to discard information pertaining to location — the so-called Visual Word Form Area (VWFA). The contribution of our article is to explain this phenomenon in a principled manner, using computational modeling. The gist of our account is that the VWFA starts out with location information, which is indeed progressively discarded as the region maturates but only in as much as actually required to recognize words presented at different retinal locations (a necessary feat when one learns how to read). This “lazy VWFA” account captures many of the findings reported by Rauschecker et al. in a simple model with very few parameters, and it makes specific predictions that would falsify the model immediately were they to be found incorrect.

## Introduction

Until recently the undisputed agreement amongst essentially all researchers in the field of visual word recognition, the current authors included, was that the Visual Word Form Area (VWFA hereafter, [1]) is a location-invariant area: that it is the seat of a computing device for “word form” representations whose mechanism —while still unknown— abstracts away from irrelevant properties such as location (see for instance [2]–[4]). This view was more than just a default prior consistent with the locus of the VWFA in the left fusiform gyrus, far down in the ventral visual processing stream. It was also suggested by analogy with the most successful hierarchical network models of invariant object recognition [5], which systematically claim location-invariance in their top layers [6], [7]. It thus came as some surprise when Rauschecker et al. demonstrated that a “blind” classifier was indeed able to categorize, with high accuracy, BOLD activation patterns evoked in the VWFA into the locations at which they had been seen by the subject [8]. Although the notion of a location-sensitive VWFA had been previously evoked by one early fMRI study [9], which explicitly manipulated word location and found support for a posterior-to-anterior gradient of sensitivity in the VWFA, the study of Rauschecker et al. is inconsistent with this account because at least in some subjects, both the posterior and the anterior portions of the VWFA were found to be sensitive to (opposite) locations in the visual field [8].

Why then should the VWFA be sensitive to the location of a word? Computational models ought to help shed light on this question, by showing how certain representations develop through learning. Embarrassingly enough however, there is currently no computational model that makes even so much as an attempt to capture how the VWFA is inserted within the network of brain areas described by Rauschecker et al., let alone attempting to describe the internal organization of the VWFA. But in trying to answer this question, we can do the next best thing and gain some insights from a class of computational models of location invariant word recognition [10]–[12]. These models all consist in a simple feedforward network that learns to recognize words independently of where they have been presented on the input space (in this article, a two-dimensional input space). Thanks to their simplicity, these models have been analyzed [13] and studied in a number of variants with an english or a french vocabulary, words of different lengths, and different visibility assumptions.

**Figure 1:**
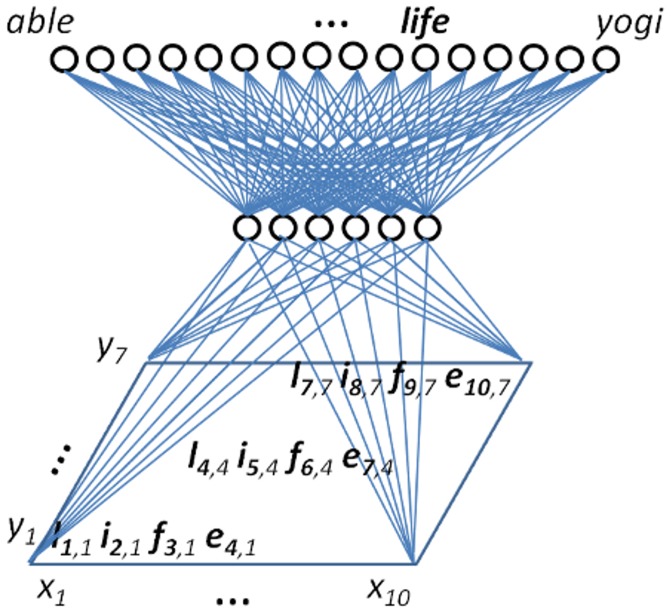
Architecture of the model. English 4-letter words were presented stochastically on a 2-dimensional input layer, and the network learned to associate the same word seen at different locations to the same abstract word unit in the output layer.

The network presented in Figure 1 is the latest instantiation of this class of models. It is not designed to investigate such questions as the role of feedback or of hemispheric integration in reading, and focuses exclusively on how location invariant word recognition might be achieved. The input layer is a location specific bank of letter detectors that codes for the presence of letters at specific horizontal and vertical locations, which would be consistent with any retinotopically organized region or group of regions between V2 and VO in the network described by Rauschecker et al. The hidden layer is where the code for any visual word stimulus —or any visual nonword stimulus— is computed, and thus it is functionally equivalent to the VWFA. The output of the network consists in a bank of location invariant word units, one for each word in the vocabulary, that may be usefully construed as word meaning representations in the pars triangularis of Broca's area (i.e. a location invariant area that receives connections from the VWFA). Every unit in the hidden layer/VWFA is assumed to receive connections from every input unit and to send connections to every output unit, initially with randomly weighted connections. Under the influence of the backpropagation learning algorithm [14], the network learns to change these connections in order to associate location specific letter inputs (e.g. 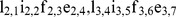) to a location invariant output (e.g. LIFE).

Our simulation procedure is described in the “Models” section, and followed in its principal lines the study of Rauschecker et al., whose material consisted of 4 letter English words and who used a linear classifier to sort activation patterns from V1/V2 and the VWFA, evoked by word stimuli presented at 6 possible locations along the horizontal and vertical axes, into 2 or 6 target locations. After training the network to recognize a vocabulary of 100 english 4-letter words presented at 7×7 possible locations, we likewise presented word inputs at 6 possible locations along the horizontal and vertical axes and collected either the input patterns or the hidden patterns (which in the rest of the article are respectively being compared to the human data for V1/V2 and for the VWFA). These patterns were then fed to a linear classifier who learned to classify them either in 2 location categories or in 6, and was tested on its ability to generalize to new patterns.

## Results

### Simulating location sensitivity in expert readers

Table 1 reports the average classification performance on input patterns and hidden patterns from all trained networks, when words were presented horizontally or vertically and when patterns were classified either into 2 or 6 classes. Let us first consider the performance of the classifier for 2 location classes.

**Table 1:** Location information in mature word representations.

	Input Layer				Hidden Layer			
	*2-Class.*	*6-Class.*	*Adj.*	*Adj. Index*	*2-Class.*	*6-Class.*	*Adj.*	*Adj. Index*
**Horizontal**	100.0% (0.0)	93.3% (3.7)	100.0%	0.98	79.8% (1.6)	34.0% (1.4)	49.5%	0.19
**Vertical**	100.0% (0.0)	100% (0.0)	57.1%	1.00	77.1% (1.5)	57.3% (2.9)	44.5%	0.36

Mean generalization accuracy of the linear classifier on input and hidden layer word representations, after training of the model (Standard error of the mean in parenthesis). Patterns corresponded to horizontally and vertically presented words, and were classified into 2 or 6 locations. For the 6 target locations classifier, percent classification to adjacent locations (Adjac.) are provided along with an index of adjacency effect (Adj. Index).

For two target classes, input representations were classified with perfect accuracy and in a way that mimics performance on human V1/V2 BOLD patterns with horizontally presented words (model 100%, human V1/V2 93%, chance 50%) and vertically presented words (model 100%, human V1/V2 92%, chance 50%). Performance for hidden representations, while overall inferior, followed the same pattern. Classification accuracy remained well above chance and at almost identical levels for horizontal and for vertical representations, which again compares well to the human data (horizontal model 79.8%, horizontal VWFA 76%; vertical model 77.1%, vertical VWFA 74%), and establishes that just like expert human readers, the trained model is location sensitive both at the input and hidden layer along the horizontal and vertical directions.

Classification performance for six location classes is potentially the most interesting, as it allows for a more fine-grained assessment of the location information present in word representations. For word representations in the input layer, performance was almost at ceiling along the horizontal and vertical axes (horizontal input layer 93.3%, vertical input layer 100%, chance 16.7%). This should be compared to classification performance on V1/V2 fMRI signals in humans, which while above chance was clearly less important than in the model (horizontal V1/V2 66.7%, vertical V1/V2 76.0%). However the observed pattern of results was very similar between humans and model in three respects: first in that they both showed a superior location sensitivity along the vertical axis, second because more classifications were made on locations adjacent to the target than to non-adjacent locations, and third because this adjacency effect was stronger along the horizontal axis than the vertical one (horizontal model 100%, horizontal human 56.3%; vertical model 57.1%, vertical human 40.6%).

Classification scores were weaker for hidden representations but still largely above chance, and location sensitivity was superior along the vertical axis than along the horizontal one (horizontal hidden layer 34.0%, vertical hidden layer 57.3%, chance 16.7%). This again mirrored qualitatively what is observed in humans (horizontal VWFA 26.2%, vertical VWFA 31.2%), including the fact that classifiers made more misclassifications on adjacent locations (horizontal model 49.5%, horizontal human 52.9%; vertical model 44.5%, vertical human 49.1%). We note that the superior strength of classification signals along the vertical axis in the model could explain why detecting adjacency effects in the VWFA for horizontally presented words is hard to achieve: the horizontal signal would be lost much faster as a function of white noise in the hidden patterns than the vertically presented signal.

**Figure 2:**
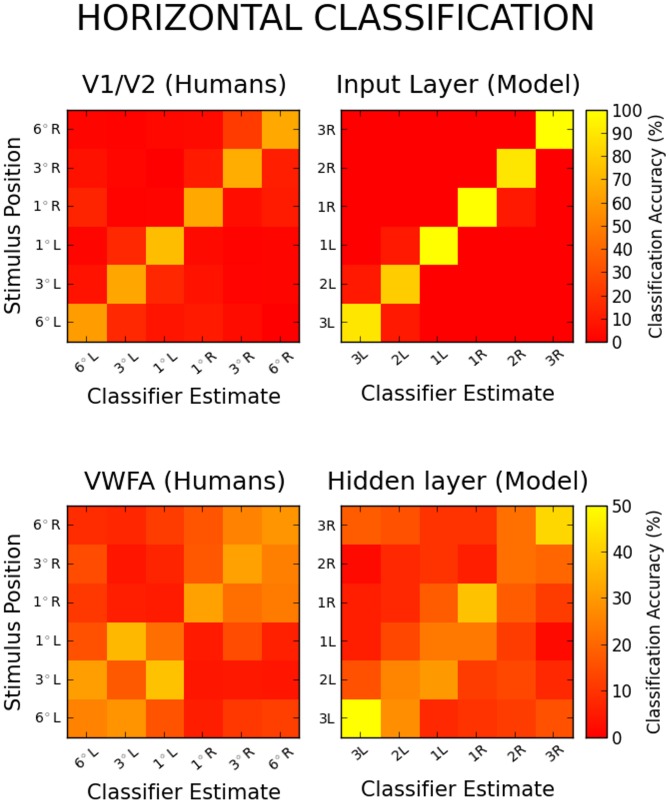
Confusion matrices for the horizontal classification of human fMRI patterns evoked in V1/V2 (Upper left) and in the VWFA (Lower left). The matrices show the average locations estimated by the classifier as a function of the actual word location (adapted from [8]). Confusion matrices for the model's input (Upper right) and hidden representations (Lower right) are averaged over 10 networks and 40 classification trials.

**Figure 3:**
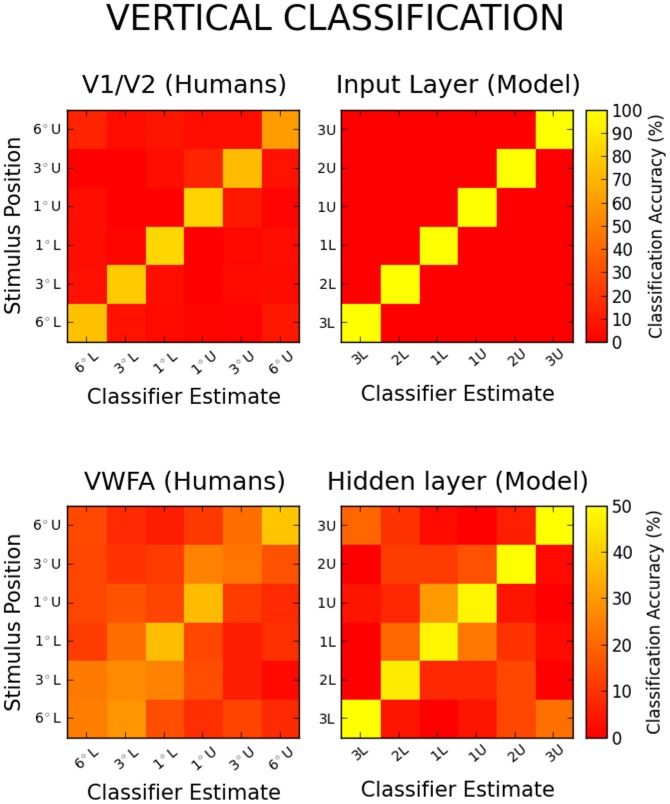
Confusion matrices for the vertical classification of human fMRI patterns evoked in V1/V2 (Upper left) and in the VWFA (Lower left). The matrices show the average locations estimated by the classifier as a function of the actual word location (adapted from [8]). Confusion matrices for the model's input (Upper right) and hidden representations (Lower right) are averaged over 10 networks and 40 classification trials.

Figures 2 and 3 provide a visual comparison between our simulations and the human data obtained by Rauschecker et al., for horizontally and vertically presented words, respectively. The agreement between model and human data is generally good, with a few visible discrepancies. Both Figures 2 and 3 (upper pannels) show that input classification is too good in the model as compared to V1/V2. Although some white noise was introduced in classified patterns in order to acknowledge the imprecision of fMRI measurements, this parameter was not fitted to the data, and increasing noise could bring input classification to the same level of performance. The model also brings support to the idea that increased classification accuracy on more central locations could be a byproduct of cortical magnification [8]. Indeed Figures 2 and 3 (lower pannels) show that this secondary phenomenon could not be reproduced in a simple model without cortical magnification, and if anything the opposite is observed as the more peripheral locations are the best classified. Finally, fMRI patterns are slightly more “hemifield specific” when elicited horizontally than vertically, with visibly less misclassifications being made across visual fields in Figure 2 than in Figure 3. This aspect of the data is beyond the scope of our single-hemispheric model.

Although the proportion of adjacent misclassifications helps to convey how similar word patterns are as a function of stimulus location, we also attempted to quantify this similarity more precisely by introducing an index that returns 0 for chance classification (no adjacency effect, a uniform confusion matrix) and increases as the distance between the actual and guessed locations decreases, to reach 1 for perfect classification at all locations (i.e. perfect classification and an identity confusion matrix, see Model section). Roughly speaking the adjacency index (AI) behaves like the geometric mean of the general classification accuracy and the adjacency of the misclassifications. Confusion matrices for the input patterns had a AI of 0.98 along the horizontal axis and 1 along the vertical axis, against 0.66 and 0.47 respectively for humans in V1/V2. Confusion matrices for hidden patterns had smaller AIs, reaching 0.19 for horizontal patterns and 0.36 for vertical patterns, to be respectively compared with AIs of 0.36 and 0.27 for humans in the VWFA.

According to the inference that high adjacency effects constitute evidence for an underlying retinotopy in the classified patterns, these results should imply a retinotopic organization of both the input and the hidden layer in the model along the vertical and horizontal axes. This might come as some surprise to the reader because although the input layer in the model is, by construction, retinotopically organized, units in the hidden layer have no contiguity: unlike the input layer, the hidden layer is a simple bag of units, and because each unit has the same total receptive field over the input layer, there can be no induced topology. Therefore this layer cannot be retinotopic in the accepted sense that contiguous units should code for contiguous inputs, and we must conclude that retinotopic organization is not the only way to account for the adjacency effects reported by Rauschecker et al. We will return to the significance and interpretation of these results in the discussion section.

### Location sensitivity across training epochs

**Figure 4:**
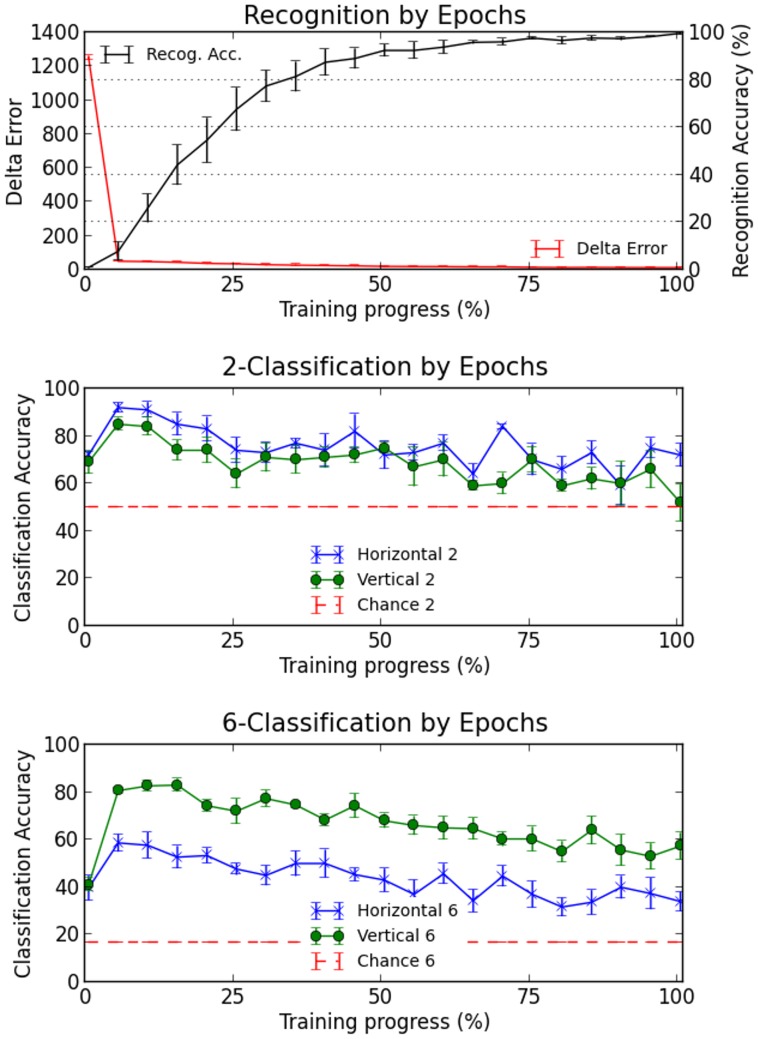
Word recognition accuracy and classification accuracy on 2 and 6 locations, for horizontal and vertical patterns, at 20 milestone epochs regularly spaced throughout training.

Figure 4 shows the average evolution of recognition accuracy and location sensitivity in 10 networks, as assessed at 20 epochs of training (to be called steps hereafter, referring to steps rather than directly to epochs is necessary given that networks needed different numbers of epochs to reach criterion: if network X took longer to train than network Y, one training step for X contains more epochs than one training step for Y). At each step location sensitivity was measured just as before, by the generalization performance of a linear classifier on sorting 40 randomly chosen hidden network patterns into 2 or 6 location classes.

Two aspects of the data are immediately obvious. First, before training (step 0) networks have as expected no recognition ability, but nonetheless they already exhibit location sensitive hidden representations, a sensitivity which peaks after the first weight modifications. From the first step onwards, the situation then reverts as networks slowly give away location sensitivity in exchange for word recognition performance. However by the time networks have met the task's requirement on word recognition, not all location sensitivity has been lost and classification accuracy is still well above chance in all conditions. Second, it can be seen that the same interaction previously found at the end of training, between the mode of presentation (horizontal or vertical) and the mode of classification (into 2 or 6 classes), extends throughout training. Specifically, performance is equally good for horizontal and for vertical patterns only when classifying into 2 classes, but much better for vertical patterns when classifying into 6 classes.

### Location sensitivity and vocabulary size

The fact that location sensitivity in the model varies inversely with recognition accuracy strongly suggests that it could be linked to the extent of the acquired vocabulary. In other words, hidden representations would end up being less and less location specific for training sets of increasing sizes. To verify this claim, we generated 50 new networks that were identical in everything but for their random initial connectivity and the training regimes they received. 10 networks were assigned to each of 5 different training regimes that used increasingly large training sets (50, 100, 150, 200 and 250 words in our simulation). As in our previous simulations, the hidden patterns for 40 randomly selected words were then collected for each of the 50 networks along the horizontal and vertical axis, and subjected to 2 dedicated classifiers that categorized them either into 2 or 6 classes.

**Figure 5:**
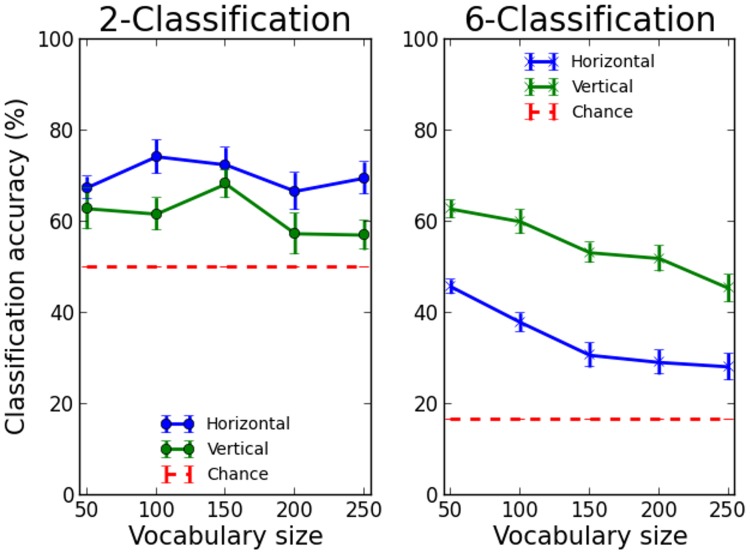
Classification accuracy as a function of network load (Lexicon size), averaged over 10 networks with random initial weights, for hidden patterns corresponding to vertically or horizontally presented words and when classified into 2 or 6 location classes.

The results are presented in Figure 5. The same general pattern of location sensitivity is found as in previous simulations: location sensitivity being everywhere above chance and showing an interaction between mode of presentation and classification type. Critically however, the results confirm that location sensitivity in the model decreases with vocabulary size as assessed by classification accuracy for 6 location classes. Although classification accuracy for 2 classes did not significantly vary across vocabulary size, the more sensitive classification accuracy for 6 classes exhibits a clear linear decrease in generalization performance from a vocabulary of 50 words (mean horizontal accuracy = 45.7%, mean vertical accuracy = 62.8%) to a vocabulary of 250 words (mean horizontal accuracy = 28.2%, mean vertical accuracy = 45.43%). This establishes that networks give up more location specificity as the vocabulary load increases, which translates into the prediction that readers with a larger estimated vocabulary should have statistically less location-specific representations in the VWFA.

The predictions we have established so far on the time-course of learning and the mature vocabulary size were derived from general learning properties of the network, by averaging classifier performance across random samples of words. But because due to their different confusability, not all the words in a training set are equally easy to learn, one would expect location specificity to be an item-level property. In a last simulation we therefore resort to an other way of testing the model at the item level, by varying the proportion of highly confusable words in the training set.

### Location sensitivity for normal words and for anagrams

In a task of location invariant word recognition, anagrams are expected to be the most difficult items to classify: whereas non-anagrams can be recognized just by the set of letters they are made of and without recourse to positional information, anagrams cannot. More generally, distinguishing between two anagrams at several locations would in average require to overcome more interference from shared letters at the same location than when distinguishing between two non-anagrams (even if they share some letters). One would then expect the network to assign more location specific representations to anagrams. This idea is put to the test in a third simulation, where 10 new networks were trained on a lexicon specially designed with 50 normal words and 50 anagrams. As previously, networks had random initial weights and were trained until convergence to criterion. Classification was then performed on horizontally or vertically presented patterns, into 2 or 6 location classes. Unlike the previous simulations however, two different linear classifiers were used to operate respectively on anagram and non-anagram patterns.

**Figure 6:**
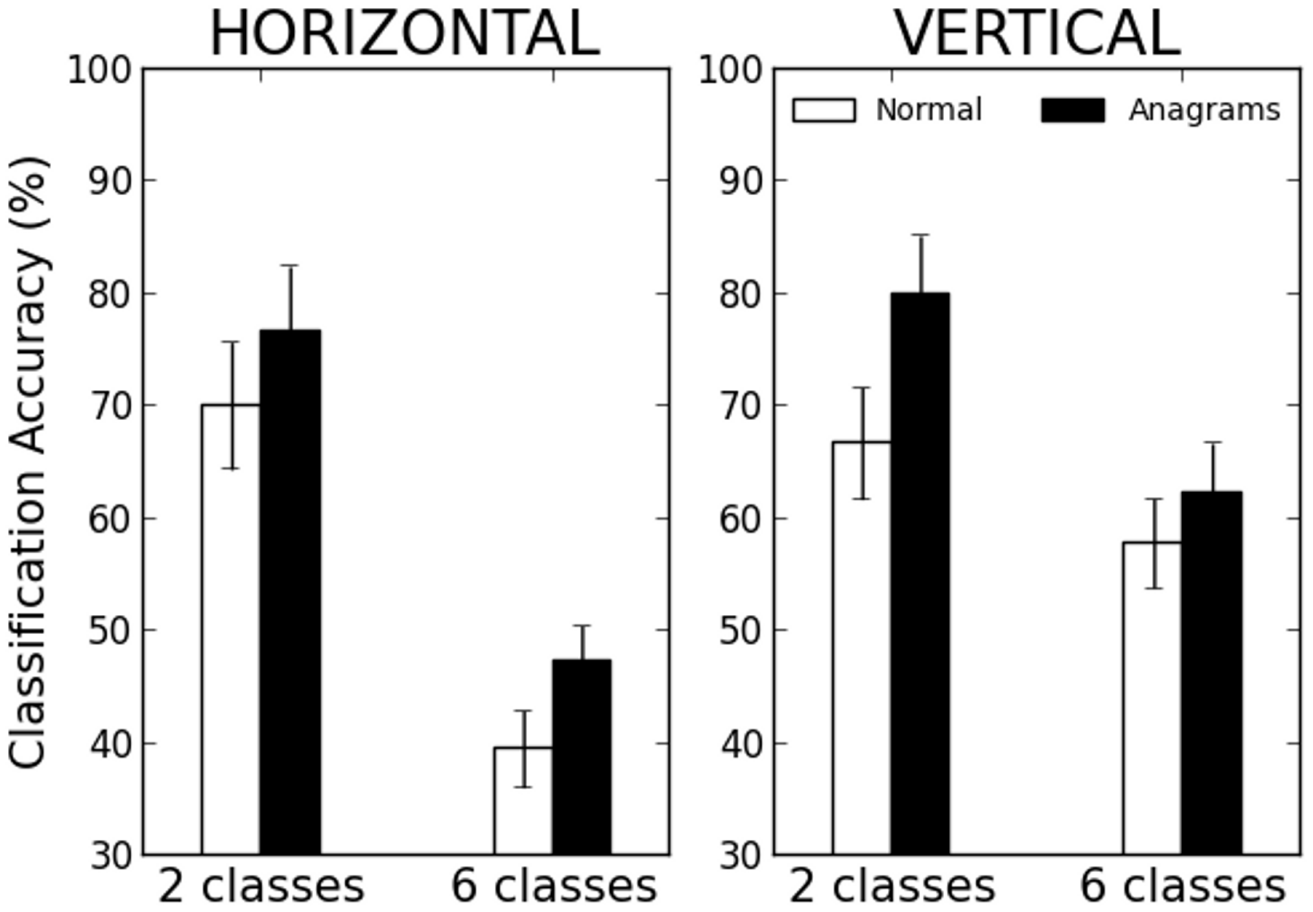
Classification accuracy for normal words and anagrams, on horizontally and vertically presented stimuli, when classified into 2 or 6 location classes.

Figure 6 illustrates the results. Classification accuracy on normal words revealed exactly the same location sensitivity pattern as previously found: accuracy was well above chance, and there was an interaction between mode of presentation and mode of classification, location sensitivity being marginally higher for horizontal patterns when sorted into 2 location classes (mean horizontal accuracy = 70.0%, mean vertical accuracy = 66.7%), but much higher for vertical patterns when classified into 6 classes (mean horizontal accuracy = 39.4, AI = 0.19; mean vertical accuracy = 57.7, AI = 0.40). Location sensitivity for anagrams, while following generally the same pattern, was higher than for normal words for 2 classes (mean horizontal accuracy = 76.7, mean vertical accuracy = 80.0) as for 6 classes (mean horizontal accuracy = 47.2, AI = 0.63; mean vertical accuracy = 62.2, AI = 0.58). Note that the adjacency index penalizes nonadjacent classification errors and therefore does not always follow the direction of mean accuracies: adjacency is marginally higher along the horizontal axis than the vertical one, despite a strong difference in mean accuracies that goes in the opposite direction. This simulation brings support to the idea that location sensitivity is an item-level property in the model, and makes the prediction that at least in adults, classifying VWFA activation patterns for anagrams should be easier than for normal words.

## Discussion

The model's agreement with experimental data would be less impressive if it had been obtained at the expense of fitting a long list of parameters, or making implausible hypotheses. It would also not be informative of the phenomenon of location-sensitivity in the VWFA if it couldn't explain the reason for it, or if it couldn't make new predictions. In what follows we examine how the model fares in all these respects.

### Making the right simplifications

The starting point of this class of models is that the VWFA is engaged in recognizing words in the face of stochastically located inputs, and that using a minimal feedforward network, one can ask how the system solves this task at the exclusion of all others and still get meaningful insights. The approach has proved successful in the past and here we have described an instance of the model that succeeds quantitatively in reproducing several surprising results of Rauschecker et al. To do this we assume only that a supervised learning algorithm is mapping location specific letter patterns produced by normally distributed word stimuli into abstract word representations. Let us look at these practical hypotheses one by one.

There is good evidence for location specific letter representations coming from behavioural and fMRI studies [9], [15], although fMRI data points to the posterior VWFA itself for the locus of these detectors. We would simply note that the complexity of the input code does not appear to play any role in the evolution of location sensitivity in the model, and one would expect the same results with less integrated inputs such as location specific letter features, or with more complex inputs such as location specific letter combinations. Indeed according to the model the critical characteristic of the input is its location specificity: it is this property which, together with random initial connection weights, ensures that the VWFA will start in a location sensitive condition. That location specific inputs should be present from the very early stages of reading is not necessarily in tension with the previously acquired location invariant object recognition skills, or with the mastering of the alphabet by children before they start to read. The general question of how children operate the transition from recognizing single letters to recognizing words is, to this day, an open-problem, but it is conceivable that children achieve this feat by harnessing intermediate stages in the letter recognition system where representations are still location specific (for instance the above mentioned location specific letter features).

The hypothesis of stochastically distributed training inputs is demanded by the well-documented stochastic component of ocular saccades during reading (see [16] for a review) as by the existence of a preferred viewing position [17] —if not simply by fixational eye movements such as microsaccades, which are correlated with bursts of neural activity in the early visual system [18]. Although the lopsided 2D-normal distribution we have used for inputs is numerically arbitrary, it is qualitatively conservative given the horizontal direction of reading in English, from which one would expect much less vertical than horizontal variance in eye fixations. As for the modeling choice that only the central location should be trained to 100% accuracy, it is supported by the fact that the probability of refixation to a given word increases with the distance of the actual landing site to the preferred viewing position [19], showing that expert readers are not trained to achieve perfect recognition for all positions. Although no data has been gathered on variability in fixations along the vertical axis, it is unrealistic to assume that there would be less refixations along the vertical axis than there are along the horizontal one. For these reasons the training protocol that uses normally distributed training patterns with a distribution lopsided on the x-axis and where only recognition at the central location was required to reach perfect scores, appears to us to be the most conservative.

Another modeling choice that should be discussed here is the absence of hemifields and hemispheres in the model. The main rationale behind this choice is that, as intriguing and significant as are the findings of Rauschecker et al. pertaining to the right homologue of the VWFA, these were not obviously relevant to the phenomenon of location sensitivity, which is the main focus of the present work. Our modeling choice therefore should be seen as a simplifying assumption rather than as reflecting a strong theoretical statement. As we have indicated, our interpretation is that the model is operating exclusively in the left hemisphere, with early integration of information coming from the right hemisphere resulting in the activation of all adequate location specific letter detectors, even if the corresponding letters were initially perceived in the left hemifield.

Finally the use of the backpropagation algorithm in a single hidden layer network might be seen as an efficient shortcut for more plausible learning algorithms operating over deeper, hierarchically organized networks. Critically, the property exhibited by our model to learn identical weights for the same letters at different locations appears not to be limited to backpropagation or to visual words, as it has recently been mirrored on a non-linguistic training base and with the Trace rule, a hebbian learning rule with a temporal window [20].

### Explanatory power

Having defended the model's assumptions, let us consider the account it gives of the phenomena described by Rauschecker et al. According to the model, the VWFA starts with representations that are location-specific and display adjacency effects, but are not at all selective for visual words. In our simulations this initial blindness to identity but sensitivity to location is reflected by word recognition being initially absent, while the classifier is still able to sort hidden word patterns by location (see Figure 4), and classified patterns have a large adjacency index along both axes.

In the model this is a consequence of the initially random connection weights afferent to the VWFA, which will conserve the location-specificity of its inputs. Adjacency effects in the hidden layer are the product of the retinotopy of the hidden layer, as well as of the differential training exposure. The fact that adjacency effects can be observed in a hidden layer that doesn't have any topology demonstrates that adjacency effects cannot be taken as a marker of retinotopy: although a retinotopic organization must imply adjacency effects, the converse does not necessarily hold.

Hidden patterns in the model therefore already start out location sensitive, being a product of location specific inputs propagated by random connection weights. But our simulations also show that the weight modifications at the very first epoch of training produce a burst of location sensitivity. This instantly brings classification performance on hidden patterns to peak values, from which they will then decrease slowly over the course of learning. It is well-known that in backpropagation networks the early weight modifications tend to be the strongest, since they are proportional to the error, thereby explaining the observed burst [21]. But it is perhaps not straightforward why this should go in the direction of more location sensitivity rather than less, or why location sensitivity should slowly decrease from the first epoch to the last. Analyses of previous instances of the model show that during training, backpropagation solves the task of location invariant word recognition by trying to assign the same weights to the same letter inputs seen at different locations [13]: in other words the model slowly turns into a symmetry network [22], and as it does so it naturally looses location sensitivity. However this does not explicitly require to destroy all information about where a word was presented, and therefore the mature representations still exhibit location sensitivity. This is a fortiori true for items that cannot be sorted out simply by considering letter identities, such as anagrams, for which letter representations will need to remain more location specific. In this view, the results of Rauschecker et al. obtain because, at least when it comes to location invariance, the VWFA chooses the path of least effort.

### Drawing predictions

Apart from providing a principled explanation of location sensitivity and adjacency effects, we see that this “lazy VWFA” account makes three testable predictions. A first prediction is that fMRI activation patterns in the VWFA should be less location specific for adults than for first-grade children, who are in the process of learning to read (see Figure 4, decreasing location sensitivity over training steps). A second prediction is that word patterns should be harder to classify in subjects with a higher estimated vocabulary (see Figure 5). Finally, a third prediction is that word patterns should be better classified when they are evoked by anagrams (see Figure 6). These predictions appear to be unavoidable in the sense that they fall out directly of the account itself, and that we expect that none of the few parameters of the model —learning rate, number of hidden units, variance of the input distribution— could be manipulated to change them. More predictions may be derived, especially concerning the impact of lexical frequency and neighborhood on location sensitivity. Although new simulations with training sets varying along these two factors would be required to draw firm predictions, from the observed impact of exposure and of letter overlap we expect that word frequency and neighborhood should be respectively negatively and positively correlated with location sensitivity.

### Conclusions and prospects

We have presented a simple learning account of location sensitivity in the VWFA, whereby maturation in this brain area is seen as a process of finding the minimal departure from an initially location sensitive connectivity, that could eventually achieve invariant word recognition. The model reproduces experimental data under parsimonious assumptions, helps to clarify some of the original data interpretations, and allows us to make testable predictions. It is also notable that none of the hypotheses we have made in this model –namely the existence of stochastically distributed retinotopic inputs, random initial connectivity, and an incremental error-correction learning algorithm– are a priori specific to visual words, and therefore a similar learning account may apply to other types of visual expertise.

**Figure 7:**
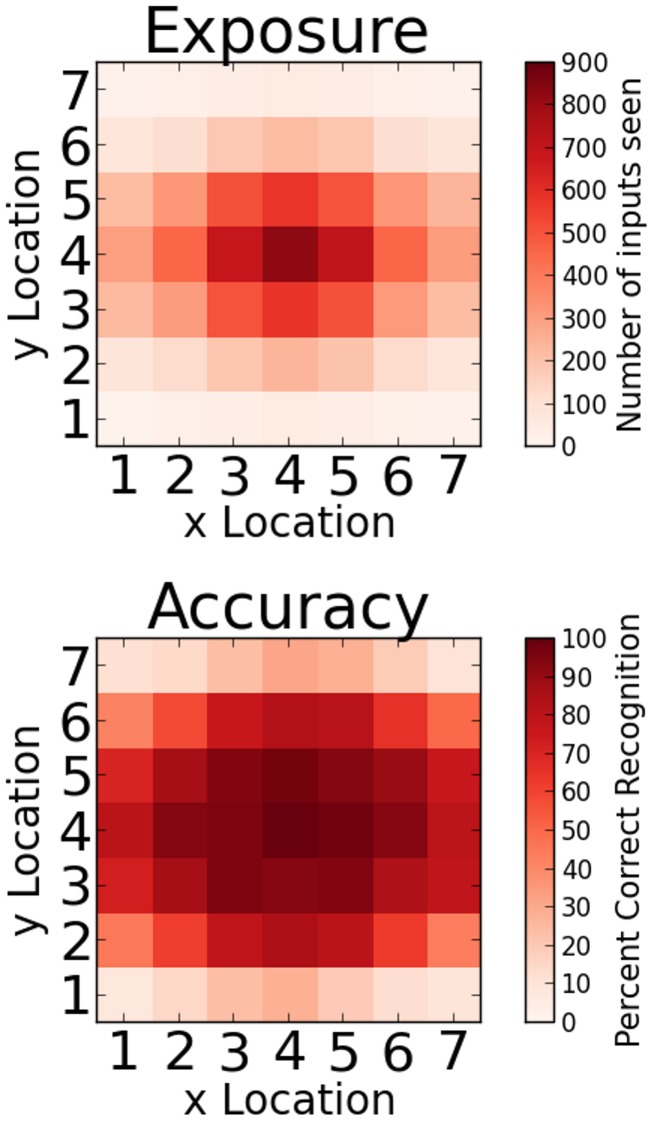
Cumulated word exposure and final recognition accuracy of the network across all input locations.

Several new analyses should be carried out to elucidate the experimental data reported by Rauschecker et al. For one thing and if the VWFA is to serve any purpose whatsoever, the activation patterns of its word exemplars ought to be better classified by identity than by location. Figure 7 suggests that this is indeed the case in the model, since by the end of training the model achieves very good recognition accuracy on all sufficiently exposed locations, which is exactly equivalent to a linear classifier like a perceptron network producing high scores on classifying hidden word representations by identities. Although the percentage of correct classification by identity is not to be found in Rauschecker et al.'s article, it would serve as a simple but critical validation of the approach. The sparseness of BOLD signals evoked by visual words, as defined for instance in [6], could also be usefully contrasted with the representations that are used in widespread and neurally inspired computational models that deal with location invariant object recognition [6], [7]. Finally if the model we have presented turns out to be warranted by subsequent studies, an instructive future step would be to address the laterality questions raised by Rauschecker et al., by running the same computational analyses on a model that explicitly distinguishes between left and right hemispheres — as for instance in [10] (see also [23]). Using two distinct hidden layers that would stand for the VWFA and its right homologue, one could hope to gain insights as to whether and how the “complementary” character of location information that Rauschecker et al. reported in these regions could indeed develop, and how it would interact with cross-hemispheric connectivity.

## Model

The model is a standard three-layer feedforward network trained under the backpropagation algorithm [14]. It has 70 location banks (10 horizontal times 7 vertical locations) of 26 letter units as an input layer, each sending connections to 50 hidden units, which in turn are fully connected to N word units in the output layer (N varied throughout the simulations, from 50 to 200 words). Initial connection weights were randomly drawn from a uniform distribution with support [−0.5,0.5]. Using random initial weights is standard practice in connectionist modeling, Input and output layers use a localist coding scheme, whereby one and only one unit stood for a given word (or a given letter/location). While input units were clamped to binary values during stimulus presentation, all other units 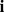 computed their activation 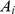 as a function of the net input 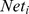 they received, using a standard sigmoid function 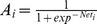. The model was trained using a vocabulary of N words on the task of associating sequences of letters seen at different locations (e.g. 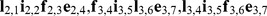) to invariant word units (e.g. **life,file,life**).

One epoch of training consisted in presenting every word in the training base exactly once. Although all input words were therefore seen with equal frequency, their locations were not uniformly distributed, but were randomly chosen anew every 5 epochs following a gaussian distribution centered on location x = 4,y = 4 (the central location), with a larger spread along the horizontal axis (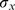 = 2.5) than the vertical one (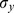 = 1.5), as shown in Figure 7 (top). Networks were trained for as many epochs as necessary to achieve perfect recognition within a radius of the central location. Unlike in previous instances of the model, for plausibility and also for convenience and speed of simulations the radius was chosen to be one in all simulations (perfect recognition was only demanded at the central location). Even with this relaxed criterion however, by the end of training a large measure of location invariance has been obtained for every word in the training set and in a way that was proportional to exposure (see Figure 7 (bottom)). For every simulation, data analysis was carried out on 10 networks instantiated with different initial weight conditions.

Once a network had been trained successfully, we randomly selected 40 words from its vocabulary and fed either their corresponding input patterns or their hidden activation patterns, obtained at the locations of interest, to a linear classifier. The locations were 6 vertical locations centered horizontally, and 6 horizontal locations centered vertically, emulating the 12 presentation conditions of Rauschecker et al. A constant amount of white noise (mean = 0.0, variance = 0.025) was also added to the patterns before classification, in order to acknowledge noise in fmri recordings (if only qualitatively). The classifier was a simple linear perceptron network, a single input layer fully connected to a single output layer with L units, and initial connection weights randomly and uniformly chosen between −0.1 and 0.1. It was trained using the delta rule (learning rate r = 0.0001) for 500 epochs on all but 6*L of the selected items, either to classify patterns in one of two location categories for C = 2 (which depending on the condition would correspond to “left” or “right”, or to “up” and “down”) or when L = 6 to classify patterns precisely into the 6 locations. In a generalization phase, we used the remaining 6*L items to test the classifier's ability to categorize new patterns. The random word selection, training and testing of the classifier were repeated for 10 runs.

To quantify the adjacency effect revealed by a classification with L location classes, we built an adjacency index that returns one for perfect classification (an identity confusion matrix), and zero in the case of random classification (a uniformly distributed confusion matrix). This is achieved by extracting the mean 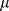 and standard deviation 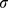 of the error distribution between guessed locations and target locations, and letting the index vary like the product of the opposite of these moments. To reflect the fact that classification errors confined to adjacent target locations reveal more adjacency than when they are randomly distributed, the index should also incorporate a contrast to the maximum standard deviation 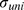 obtained in the case of a uniform distribution. An index that meets all of these criteria is:
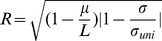
Which can be interpreted as the geometric mean of the classification accuracy and the error distribution's departure from uniformity.

## References

[1] Cohen L, Dehaene S, Naccache L, Lehéricy S, Dehaene-Lambertz G, et al (2000) The visual word form area: Spatial and temporal characterization of an initial stage of reading in normal subjects and posterior split-brain patients. Brain 123: 291307. 10.1093/brain/123.2.291 10648437

[2] Cohen L, Lehéricy S, Chochon F, Lemer C, Rivaud S (2002) Language-specific tuning of visual cortex? Functional properties of the Visual Word Form Area Brain 125: 10541069. 10.1093/brain/awf094 11960895

[3] Dehaene S, Pegado F, Braga LW, Ventura P, Nunes Filho G, et al (2010) How learning to read changes the cortical networks for vision and language. Science 330: 13591364. 10.1126/science.1194140 21071632

[4] Wandell BA, Rauschecker AM, Yeatman J (2012) Learning to see words. Annu Rev Psychol 63: 3153. 10.1146/annurev-psych-120710-100434 PMC3228885 21801018

[5] Dehaene S, Cohen L, Sigman M, Vinckier F (2005) The neural code for written words: a proposal. Trends Cogn Sci 9: 335–341. 15951224 10.1016/j.tics.2005.05.004

[6] Wallis G, Rolls ET (1997) Invariant face and object recognition in the visual system. Progress in Neurobiology 51: 167–194. 9247963 10.1016/s0301-0082(96)00054-8

[7] Serre T, Oliva A, Poggio T (2007) A Feedforward Architecture Accounts for Rapid Categorization. Proc Natl Acad Sci USA 104 ((15)) 6424–6429. 17404214 10.1073/pnas.0700622104 PMC1847457

[8] Rauschecker AM, Bowen RF, Parvizi J, Wandell BA (2012) Position sensitivity in the visual word form area. Proc Natl Acad Sci USA 109 ((24)) 1568–1577. 10.1073/pnas.1121304109 PMC3386120 22570498

[9] Dehaene S, Jobert A, Naccache L, Ciuciu P, Poline JB, et al (2004) Letter binding and invariant recognition of masked words: Behavioral and neuroimaging evidence. Psychol Sci 15: 307313. 10.1111/j.0956-7976.2004.00674.x 15102139

[10] Shillcock RC, Monaghan P (2001) The computational exploration of visual word recognition in a split model. Neural Computation 13: 1171–1198. 11359649 10.1162/08997660151134370

[11] Dandurand F, Grainger J, Dufau S (2010) Learning location invariant orthographic representations for printed words. Connection Science 22 ((1)) 25–42.

[12] Dandurand F, Hannagan T, Grainger J (2013) Computational Models of Location-Invariant Or- thographic Processing. Connection Science 25 ((1)) 1–26.

[13] Hannagan T, Dandurand F, Grainger J (2011) Broken Symmetries in a Location-Invariant Word Recognition Network. Neural Computation 23 ((1)) 251–283. 20964541 10.1162/NECO_a_00064

[14] Rumelhart DE, Hinton GE, Williams RJ (1986) Learning representations by back-propagating errors. Nature 323 ((6088)) 533536.

[15] Peressotti F, Grainger J (1999) The role of letter identity and letter position in orthographic priming. Perception & Psychophysics 61: 691–706. 10370337 10.3758/bf03205539

[16] Rayner K (2009) Eye movements and landing positions in reading: a retrospective. Perception 38 ((6)) 895–899. 19806981 10.1068/pmkray

[17] Rayner K (1979) Eye guidance in reading: fixation locations within words. Perception 8 ((1)) 21–30. 432075 10.1068/p080021

[18] Martinez-Conde S, Macknik SL, Hubel DH (2004) The role of fixational eye movements in visual perception. Nat Rev Neurosci 5: 229–240. 14976522 10.1038/nrn1348

[19] O'Regan JK, Lévy-Schoen A (1987) Eye movement strategy and tactics in word recognition and reading. In M. Coltheart (Ed.), Attention and performance XII: The psychology of reading, Erlbaum, Hillsdale, NJ 363383.

[20] Léveillé J, Hannagan T (2013) Learning spatial invariance with the trace rule in non-uniform distributions. Neural Computation 25 ((5)) 1261–1276. 23470122 10.1162/NECO_a_00435

[21] Smith MA, Cottrell GW, Anderson KL (2001) The early word catches the weights. In: Leen TK, Dietterich, Tresp V, editors. Advances in neural information processing systems. Cambridge, MA: MIT Press.

[22] Shawe-Taylor J (1993) Symmetries and Discriminability in Feedforward Network Architectures. IEEE Transactions on Neural Networks 4 ((5)) 816–826. 18276511 10.1109/72.248459

[23] Weems S, Reggia J (2004) Hemispheric specialization and independence for word recognition: A comparison of three computational models. Brain and Language 89 ((3)) 554–568. 15120546 10.1016/j.bandl.2004.02.001

